# Angiopoietin-like protein 3 markedly enhanced in the hyperlipidemia related proteinuria

**DOI:** 10.1186/s12944-019-1052-1

**Published:** 2019-05-18

**Authors:** Xia Gao, Yanhong Suo, Min Zhang, Yan Wang, Xin Gao, Qiu Bing, Qingju Liu

**Affiliations:** 1 Pediatric Department, Gansu Provincial Hospital, No. 204 Donggang West road, Lanzhou City, 730000 China; 2 Ningxia Medical University, Yinchuan City, 750000 China; 3 Intensive Care Unit, Gansu Provincial Hospital, Lanzhou City, 730000 China; 4 Gansu University of Chinese Medicine, Lanzhou City, 730000 China

**Keywords:** Hyperlipidemia, Angiopoietin-like protein 3, 24 h urine protein

## Abstract

**Background:**

Angiopoietin-like protein 3(ANGPTL3) is well acknowledged as a key regulator of lipid metabolism. Now, there have not been enough data to explain the mechanism of hyperlipidemia related proteinuria. In this study, we hoped to investigate the changes of Angiopoietin-like protein 3(ANGPTL3) levels in hyperlipidemia patients with different proteinuria levels.

**Methods:**

Seventy-one patients with hyperlipidemia were selected, who were hospitalized in Gansu Provincial People’s Hospital from September 2016 to September 2017, and 20 healthy people in the physical examination center were selected. We combed through medical history and conducted clinical biochemical indicators of blood urea nitrogen (BUN), serum creatinine (SCr), 24 h urine protein quantitation (24hUPro), cholesterol (TC), triglyceride (TG), high density lipoprotein (HDL) and low detection of density lipoproteins (LDL-C). The concentration of serum ANGPTL3 was measured by ELISA.

**Results:**

1. Serum ANGPTL3 in patients with hyperlipidemia related proteinuria was higher than that in the control group, and the difference was statistically significant (*p* < 0.05); 2. 24hUPro and BMI (r = 0.321, *P* = 0.002), TC (r = 0.465, *P* = 0.000), TG (r = 0.281, *P* = 0.007), LDL (r = 0.478, *P* = 0.000) in patients with hyperlipidemia related proteinuria are positively correlated, suggesting that dyslipidemia is related to the occurrence of proteinuria; 3. BMI, TC, TG and LDL in patients with hyperlipidemia related proteinuria were positively correlated with serum ANGPTL3. 4. The 24hUPro of patients with hyperlipidemia related proteinuria was positively correlated with serum ANGPTL3 levels, and BUN and SCr were not associated with serum ANGPTL3 level. 5. There was no significant difference in TC, TG, BMI, 24hUPro and serum ANGPTL3 between the statin-treated and the untreated groups in patients with hyperlipidemia related proteinuria.

**Conclusions:**

Angiopoietin-like protein 3 markedly enhanced in the hyperlipidemia related proteinuria.

## Introduction

Hyperlipidemia is becoming a common disease in China with the improvement of people’s living standards and the changes of their lifestyle. In 2002, China conducted the nationwide blood lipid epidemiological Screening, the Screening on nutrition and health status of residents. The results showed that the total lipid abnormality rate of Chinese people over 18 years old was as high as 18.6% [1]. Alarmingly, the prevalence rate soared to 41.9% [2] in 2014, an increase of 225.3% compared to the results in 2002.

In 2014, Kidney Disease Outcome Quality Initiative (K/DOQI) proposed that hyperlipidemia was an important marker of the chronic kidney disease(CKD) progression [3], the lipid profile was an independent risk factor for CKD management. Proteinuria is a key clinical indicator for the progression of renal damage. The mechanism of the occurrence of hyperlipidemia proteinuria has not been fully elucidated.

Angiopioetin-like protein 3 (ANGPTL3), discovered in 1999, is a new member of the angiogenin family. The activity plays an important role in lipid metabolism. In recent years, studies have shown that ANGPTL3 is mainly expressed in human hepatocytes and renal podocytes. After hepatocytes secretion, ANGPTL3 inhibits lipoprotein lipase (LPL) activity [4, 5], resulting in plasma VLDL increased, triglyceride cholesterol decomposition decreased and blood lipids increased. At the same time, its involvement in angiogenesis is mainly through receptor integrin beta 3, which is considered as a key molecule in regulating lipid metabolism [6, 7].

In this study, we found that hepatocytes, glomerular podocytes could synthesize ANGPTL3 16, and its expression level is positively correlated with the degree of proteinuria and podocyte process fusion [8]. Furthermore, vitro studies confirmed that ANGPTL3 induced podocyte cytoskeletal protein rearrangement and increased podocyte motility [9]. However, whether ANGPTL3 is involved in the development of hyperlipidemia proteinuria had not been confirmed. This study focused on the characteristics of serum ANGPTL3 expression in patients with hyperlipidemia and complicated with proteinuria and its relationship with renal damage related indicators. The results of the study are reported as follows.

## Methods

### Research selection criteria

Inclusion criteria for patients with hyperlipidemia:

(1) Total cholesterol (TC) is greater than 6.2 mmol/L (240 mg/d1); (2) Triglyceride (TG) is greater than 2.3 mmol/L (200 mg/d1); (3) Low density lipoprotein cholesterol (LDL-C) is greater than 4.1 mmol/L (160 mg/d1). (4) The above 3 articles are in line with any of them, which is consistent with the 2016 Chinese adult dyslipidemia prevention guideline (Revised Edition 2016) diagnostic classification standard [5].

Exclusion criteria for patients with hyperlipidemia:

(1) detailed inquiry for medical history, physical examination and necessary laboratory tests, the diagnosis of nephrotic syndrome, diabetes, hypothyroidism, liver disease, renal failure, systemic lupus erythematosus, glycogen accumulation, myeloma, fat atrophy, acute porphyria, and more The use of certain drugs such as glucocorticoids, diuretics and other drugs that affect the metabolism of blood lipids are excluded; (2) the tumor patients in various systems; (3) patients themselves or their guardians fail in signing informed consent.

### Patients

#### Case group

Seventy one patients with hyperlipidemia who were hospitalized in Gansu Provincial People’s Hospital from September 2016 to September 2017 were selected, including 42 males and 29 females, aged from 11 to 84, with an average age of 52.73 ± 15.64 years old.

#### Control group

Twenty healthy people from the Physical Examination Center of Gansu Provincial People’s Hospital were selected as the normal control group (NC group), including 10 males and 10 females, from aged 3 to 61, with an average age of 32.50 ± 19.18 years old, excluding various diseases. The healthy control group had total cholesterol TC < 6.2 mmol/L and triglyceride TG < 2.3 mmol/L.

### Serum collection

Blood samples of patients and controls were centrifuged at 2400 rpm for 5 min. Serum was collected and stored at − 80 C until samples analysis.

### Serum ANGPTL3

Serum ANGPTL3 concentration was measured by double antibody sandwich enzyme-linked immunosorbent assay (ELISA). The kit was purchased from University College London (UCL) company, CV < 5% in batch and CV < 10% in batch, and the lowest detection limit was 75 pg/ml.

### Clinical biochemical indexes

Blood Urea Nitrogen (BUN), Serum Creatinine (SCr), 24 h Urine Protein Quantitative (24hUPro), Cholesterol (TC), Triglyceride (TG), High-density lipoprotein (HDL) and low density lipoprotein (LDL) were detected by the automatic biochemical analyzer of the laboratory of Gansu Provincial People’s Hospital.

### Statistical analysis

In this study, SPSS17.0 software was used to analyze the data. The measurement data were represented by the standard deviation (x ± s). The two groups were compared with two independent sample t-tests. The variance homogeneity test was performed by Levene method. The variance was homogeneous by single factor analysis of variance, and the variance was inhomogeneous by rank sum test. Correlation was analyzed by Spearman correlation analysis, and *p* < 0.05 was statistically significant.

## Results

### Comparison of observation indexes between hyperlipidemia group and normal control group

A total of 71 patients with hyperlipidemia were included in the study, all of which met the 2016 Chinese adult dyslipidemia prevention and treatment guidelines (2016 revised edition) diagnostic classification criteria, 20 normal control group. As shown in Table 1. the mean age of patients with hyperlipidemia related proteinuria was 52.73 ± 15.64, and that of the normal control group was 32.50 ± 19.18. The two groups were statistically significant (*P* < 0.05), suggesting that the occurrence of lipid metabolism disorder would become more serious with age increase, so early detection is necessary. The BMI of the two groups were 24.90 ± 3.91 and 20.56 ± 1.36, respectively.

**Table 1:** Comparison of observation indexes between hyperlipidemia group and normal control group $\left(\overline{x}\pm \kern0.5em s\right)$

Group	Hyperlipidemia group (*n* = 71)	Normal control group (*n* = 20)	*t*/χ^2^	*p*
Sex (male/female)	42/29	10/10	0.534	0.465
Age (age)	52.73 ± 15.64	32.50 ± 19.18	4.855	0.000
Systolic pressure (mmHg)	133.07 ± 20.38	124.30 ± 10.93	2.551	0.068
diastolic pressure (mmHg)	83.94 ± 13.07	76.45 ± 9.40	2.391	0.019
BMI	24.90 ± 3.91	20.56 ± 1.36	7.840	0.000
TC (mmol/L)	6.20 ± 2.03	3.67 ± 0.91	8.004	0.000
TG (mmol/L)	4.27 ± 3.41	1.16 ± 0.50	7.415	0.000
HDL (mmol/L)	1.29 ± 0.52	1.17 ± 0.32	1.006	0.317
LDL (mmol/L)	3.93 ± 1.72	1.85 ± 0.36	9.488	0.000
24hUPro (g/d)	1.44 ± 2.89	0.014 ± 0.017	4.417	0.000
BUN (mmol/L)	6.51 ± 4.02	5.45 ± 1.78	1.144	0.256
SCr (umol/L)	94.63 ± 141.77	51.21 ± 18.77	1.361	0.177
Serum ANGPTL3 (ng/ml)	87.86 ± 77.13	44.10 ± 32.17	2.470	0.015

The difference was statistically significant (*P* < 0.05), suggesting that BMI is an important clinical feature of hyperlipidemia. There was no significant difference in systolic blood pressure between the two groups.

Analysis of the main indicators of hyperlipidemia in the renal damage group and the normal control group showed that the average TC levels in the two groups were 6.20 ± 2.03 and 3.67 ± 0.91, respectively, and the difference was statistically significant (*P* = 0.000). The average TG levels were 4.27 ± 3.41 and 1.16 ± 0.50, respectively, and the difference was statistically significant (*P* = 0.000). The mean LDL levels were 3.93 ± 1.72 and 1.85 ± 0.36, respectively, and the difference was statistically significant (*P* = 0.000). There is no difference in HDL.

The analysis of the indexes related to renal damage in the hyperlipidemia related proteinuria group and the normal control group showed that the average 24-h urine protein levels in the two groups were 1.44 ± 2.89 and 0.014 ± 0.017, respectively, and the difference was statistically significant (*P* = 0.000); There is no difference in SCr.

The mean serum ANGPTL3 levels were 87.86 ± 77.13 and 44.10 ± 32.17, respectively, and the difference was statistically significant (*P* < 0.05). The differences of BMI, TC, TG, LDL and 24 h urine protein were highly statistically significant (*P* < 0.001). There were no differences in systolic blood pressure, HDL, BUN, and SCr between the two groups.

### Comparison of renal damage index and serum ANGPTL3 expression in patients with hyperlipidemia related proteinuria at different blood pressure levels

In this study we selected inpatients to performed case-control study. we found that patients suffering from hyperlipidemia were often accompanied by hypertension in the same time. As a member of the angiopoietin-like protein family, ANGPTL3 is involved in the proliferation of vascular endothelial cells, and whether this molecule is related to hypertension needs to be investigated. Whether hyperlipidemia patients combined hypertension will aggravate the abnormal expression of ANGPTL3 is the question to be worth paying attention to study. So, in this report 71 cases of hyperlipidemia renal damage patients were divided into hyperlipidemia with normal blood pressure group (*n* = 34) and hyperlipidemia with hypertension group (*n* = 37). The renal damage index and serum ANGPTL3 expression levels were compared between the two groups. There were no significant differences in BUN, SCr, 24hUPro and serum ANGPTL3 (Table 2) between the normal blood pressure group and the abnormal group the two groups (*P* > 0.05).

**Table 2:** Comparison renal damage index and serum ANGPTL3 of normal patients with Hypertension patients

Group	hyperlipidemia Normal blood pressure group (*n* = 34)	hyperlipidemia Hypertension group (*n* = 37)	t	*p*
BUN (mmol/L)	6.19 ± 2.14	6.80 ± 5.21	−0.637	0.526
SCr (umol/L)	69.93 ± 28.91	117.33 ± 192.89	−1.476	0.148
24hUPro (g/d)	0.78 ± 1.43	2.04 ± 3.69	−1.916	0.061
Serum ANGPTL3 (ng/ml)	94.30 ± 78.71	81.95 ± 76.25	0.672	0.504

### Correlation between blood lipid index and renal damage index in patients with hyperlipidemia related proteinuria

Previous studies have shown that hyperlipidemia could aggravate the occurrence of renal damage. This study specifically observed the relationship between blood lipid index and renal damage index of BMI, TC, TG, HDL and LDL. The correlation analysis of each observation index was shown in Table 3. BMI was positively correlated with SCr (r = 0.266, *P* = 0.011), 24hUPro (r = 0.321, *P* = 0.002), and not related to BUN; TC and BUN (r = 0.230, *P* = 0.028), 24hUPro (r = 0.465, *P* = 0.000) showed a positive correlation and no correlation with SCr; TG was positively correlated with 24hUPro (r = 0.281, *P* = 0.007), and was not correlated with BUN and SCr; HDL was not associated with BUN, SCr and 24hUPro; LDL and SCr (r = 0.338, *P* = 0.001), 24hUPro (r = 0.478, *P* = 0.000) showed a positive correlation and was not related to BUN.

**Table 3:** Correlation analysis of BMI, TC, TG, HDL, LDL results renal damage index and serum ANGPTL3

Factor	BMI		TC		TG		HDL		LDL	
	r	*P*	r	*P*	r	*P*	r	*P*	r	*P*
BUN	−0.31	0.767	0.230	0.028	−0.026	0.804	0.370	0.730	0.197	0.062
SCr	0.266	0.011	0.180	0.088	0.087	0.414	0.078	0.525	0.338	0.001
24hUPro	0.321	0.002	0.465	0.000	0.281	0.007	0.039	0.716	0.478	0.000
Serum ANGPTL3	0.367	0.000	0.233	0.026	0.395	0.000	0.062	0.557	0.293	0.005

### Correlation between serum lipid index and serum ANGPTL3 in patients with hyperlipidemia related proteinuria

We know that ANGPTL3 is a key molecule in regulating lipid metabolism. This study specifically observed the relationship between BMI, TC, TG, HDL and LDL blood lipids and ANGPTL3. The correlation analysis of each observation index was shown in Table 3: BMI and serum ANGPTL3 (r = 0.367, *P* = 0.000) was positively correlated, TC was positively correlated with serum ANGPTL3 (r = 0.233, *P* = 0.026), TG was positively correlated with serum ANGPTL3 (r = 0.395, *P* = 0.000), HDL and serum ANGPTL3 were not correlated. Correlatively, LDL was positively correlated with serum ANGPTL3 (r = 0.293, *P* = 0.005), indicating that serum ANGPTL3 expression was associated with elevated BMI, TC, TG, and LDL.

### Correlation between serum ANGPTL3 level and renal damage markers in patients with hyperlipidemia related proteinuria

**Fig. 1:**
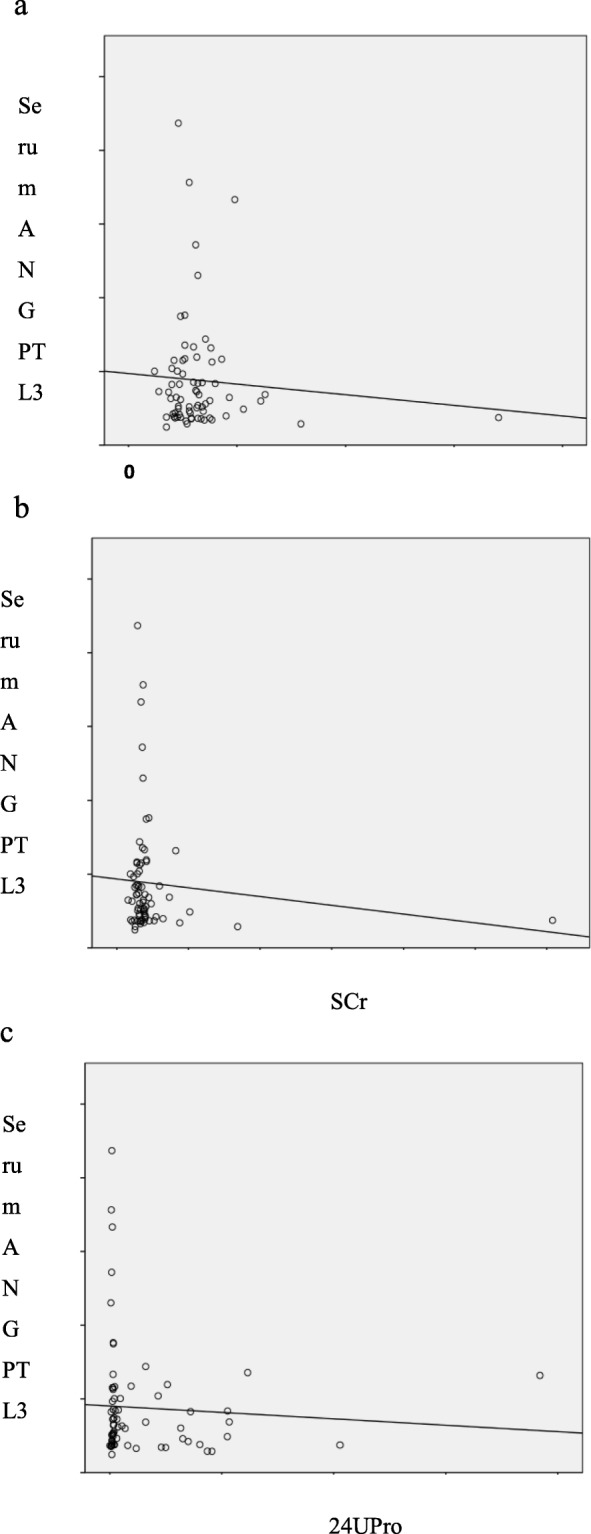
Correlation analysis of serum ANGPTL3 levels results renal damage. **a**, **b**: the expression level of serum ANGPTL3 was not correlated with BUN and SCr; **c**: the expression level of serum ANGPTL3 was positively correlated with 24hUPro level (r = 0.248, *P* = 0.018)

In order to understand the relationship between serum ANGPTL3 level and renal damage index in hyperlipidemia group, this study analyzed BUN, SCr and 24hUPro. The results showed that the expression level of serum ANGPTL3 was not correlated with BUN and SCr, but positively correlated with 24hUPro level (r = 0.248, *P* = 0.018), which can be seen clearly in Fig. 1.

### Comparison of effects of statins on serum lipids, renal damage and serum ANGPTL3 expression in patients with hyperlipidemia related proteinuria

Statins are currently used in the treatment of hyperlipidemia. We are trying to understand whether statins could improve blood lipids and proteinuria in patients with hyperlipidemia related proteinuria. As shown in Table 4, the TC of the statin-treated patients with hyperlipidemia was 6.34 ± 1.70, and the untreated group was 5.93 ± 2.56. The difference was not statistically significant. The TG of the two groups were 4.63 ± 3.99, 3.61 ± 1.83. There was no statistical significance; the BMI of the two groups was 25.04 ± 3.91, 24.65 ± 3.97,and the difference of was not statistically significant. Further analysis results of the 24hUPro of the two groups were 25.04 ± 3.91, 24.65 ± 3.97, respectively. There was no significant difference between the two groups, indicating that the statin administration did not affect the 24hUPro level of the patients; the serum ANGPTL3 of the two groups were 105.4 ± 88.51, 78.33 ± 69.37, respectively. The significance of the study indicates that taking statins has no effect on serum ANGPTL3 levels in patients with hyperlipidemia related proteinuria.

**Table 4:** After given statins, the change of serum lipid and kidney damage indicators and serum ANGPTL3 (*x*^‵^  ±  *s*)

Group	Untreated group (*n* = 25)	Treatment group (*n* = 46)	t	*p*
BMI	24.65 ± 3.97	25.04 ± 3.91	0.395	0.694
TC (mmol/L)	5.93 ± 2.56	6.34 ± 1.70	0.811	0.420
TG (mmol/L)	3.61 ± 1.83	4.63 ± 3.99	1.474	0.145
HDL (mmol/L)	1.34 ± 0.64	1.27 ± 0.44	−0.565	0.574
LDL (mmol/L)	3.99 ± 2.23	3.89 ± 1.38	− 0.218	0.828
24hUPro (g/d)	2.13 ± 4.28	1.06 ± 1.67	−1.207	0.237
Serum ANGPTL3 (ng/ml)	105.4 ± 88.51	78.33 ± 69.37	−1.423	0.159

### Expression of ANGPTL3 in different stages of chronic kidney disease in patients with hyperlipidemia

In this study, patients with hyperlipidemia were divided into the following three stages depending on glomerular filtration rate. It can be seen from the data in Table 5 that there was no significant difference in the expression level of blood ANGPTL3 in patients with different chronic kidney disease stages in the hyperlipidemia group. Difference (F = 0.745, *P* = 0.478). It indicated that blood ANGPTL3 level was positively correlated with 24 h hour urine protein, but it could not be used as an index to evaluate renal function damage.

**Table 5:** Expression of ANGPTL3 in different chronic kidney disease stages

GFR (*n*)	Serum ANGPTL3 (ng/ml)
≥90 (47)	90.15 ± 76.67
60~89 (15)	97.71 ± 95.88
< 59 (9)	59.51 ± 32.43
F	0.745
*P*	0.478

## Discussion

In 1982, the concept of lipid nephrotoxicity was first proposed by Moorhead et al [10] Lipid plays an important role not only in mediating glomerular injury, but also in tubulointerstitial injury, which could cause glomerular basement membrane thickening and renal interstitial fibrosis, finally develops into uremia [11]. However, the mechanism of renal damage caused by hyperlipidemia is unclear.

ANGPTL3 is a key molecule in regulating lipid metabolism in humans. We found that ANGPTL3 was closely related to renal damage [9]. Our reported results confirm that it was positively correlated with the degree of proteinuria in nephrotic syndrome [8]. Then, gene knockout experiments also showed that the mice did not show a large number of typical nephrotic syndromes such as proteinuria and hyperlipidemia [9], with the lack of ANGPTL3. In addition to being involved in lipid metabolism, this molecule was also involved in the damage of glomerular podocytes and was a key molecule in the development of nephrotic proteinuria.

Whether ANGPTL3 participates in the occurrence of proteinuria in patients with hyperlipidemia was our key concern. This study analyzed the relationship between ANGPTL3 and renal damage related indicators in hyperlipidemia population, hoping to provide the new ideas for revealing the occurrence of hyperlipidemia related proteinuria.

This study found that the age, BMI, TC, TG and LDL of patients with hyperlipidemia related proteinuria were significantly higher than those of the normal control group, and the difference was statistically significant. The lipid metabolism index was gradually aggravated with age which told us that hyperlipidemia was a chronic progression process. It was necessary to give intervention in early clinical stage.

Further analysis showed that the level of 24hUPro in patients with hyperlipidemia related proteinuria was significantly higher than the normal control group, indicating that patients with hyperlipidemia were more likely to suffer from renal damage than normal people.

It has been shown in animal studies that the occurrence and progression of renal damage in the context of hyperlipidemia was often accompanied by glomerular sclerosis and tubulointerstitial damage [12–14]. And Hypertriglyceridemia and hypercholesterolemia could cause podocyte injury and focal segmental sclerosis in rats [15]. Low-density lipoprotein could be oxidized by mesangial cells and stimulate pro-inflammatory and pro-fibrotic cytokines, vasoactive substances and apoptosis [16]. Vitro studies have shown that destruction of fatty acid oxidation increases apoptosis, intracellular lipid deposition and dedifferentiation of renal tubular epithelial cells [17]. In our study, BMI, TC, TG, LDL and 24hUPro were positively correlated in the hyperlipidemia related proteinuria group, which showed that proteinuria increased with the increase of BMI, TC, TG and LDL, but there was no correlation with BUN and SCr, which is consistent with the experimental results of these animals and cells, and further confirms the correlation between lipid metabolism and proteinuria. At the same time, some studies have found that patients with hyperlipidemia for more than 1 year, the urine microalbumin (ALB) had increased when urine, BUN and SCr were normal, which suggested that urine ALB could be used as early sensitive indicators of kidney damage in patients with hyperlipidemia [18]. The patients selected in this study were hospitalized patients, and there was massive proteinuria. Therefore, no urine micro-ALB was detected. Our group will pay more attention to the urine micro-ALB level in patients with community and outpatients with hyperlipidemia. The serum of patients with early renal damage in hyperlipidemia was collected for further analysis.

Recent study found that the expression of the liver ANGPTL3 was significantly increased after the high cholesterol diet of mice [19]. In our study, we also confirmed there was a positive correlation between serum ANGPTL3 and BMI (r = 0.367, *P* = 0.000), and the difference was statistically significant, suggesting that abdominal obesity was associated with ANGPTL3. The study also showed that TC, TG, LDL and serum ANGPTL3 levels were positively correlated, the difference was statistically significant. With the increase of TC, TG and LDL levels, serum ANGPTL3 levels increased, suggesting that hyperlipidemia may due to the increasing expression of serum ANGPTL3. Our results were consistent with previous studies suggesting that ANGPTL3 was a sensitive expression indicator in patients with clinical hyperlipidemia.

It was important to confirm that there was the positive correlation between serum ANGPTL3 level and 24hUPro in patients with hyperlipidemia related proteinuria by Spearman correlation analysis. The difference is statistically significant. The serum ANGPTL3 level increases with the increase of urinary protein, suggesting that ANGPTL3 might be involved in occurrence of lipemia proteinuria. The ANGPTL3 might regulate lipid metabolism, and abnormal regulation of lipid metabolism may involve the glomerular sclerosis earlier, which may induce oxidative stress in local environment under high-fat diet. And previous studies have shown that glomerular podocytes can express angiopoietin-like protein 3 (ANGPTL3). It has been found that ANGPTL3 may regulate the motility of podocytes by altering the expression of nephrin, which was involved in the occurrence of proteinuria [8, 9]. ANGPTL3 was highly expressed in patients with hyperlipidemia and may be a molecule that interferes with the occurrence of proteinuria in hyperlipidemia, which has been issued by our research group.

BUN and SCr were used as indicators of renal function tests. We found there were no correlation between BUN or SCr and ANGPTL3, which was consistent with our results in vivo cell experiments. ANGPTL3 played important role in proteinuria and may be involved in podocyte injury.

Statins have been well known as classic lipid-lowering drugs. Statins are currently the primary treatment for hyperlipidemia, but it’s efficacy for patients with hyperlipidemia related proteinuria has still been a controversial issue. Our statistics found that after the treatment of statins in patients with hyperlipidemia related proteinuria there was no statistically significant effect on serum lipids, renal damage, and serum ANGPTL3 expression levels, which may be affected by the small sample size of the early collection, the detection method and the poor compliance in patients with hyperlipidemia related proteinuria. It is known that statins reduce the production of product TC mainly by blocking the binding site of hydroxymethylglutaryl coenzyme A reductase (HMGCoA-R) to a substrate [20]. However, its protective effect on kidneys that does not rely on lipid-lowering has received attention in recent years [21]. We observed that there was no difference in the effect of statin-treated and untreated groups on blood lipids in patients with hyperlipidemia related proteinuria, which needs to be further verified in animal models in order to find an effective treatment for jeopardizing a patient’s complications both to reduce hyperlipidemia and proteinuria. The ANGPTL3 which mainly regulates lipid metabolism in the body, is thought to be a connection point of hyperlipemia and proteinuria, which may be closely related to chronic renal disease lipid metabolism and proteinuria.

## Conclusion

The expression level of serum ANGPTL3 in patients with hyperlipidemia related proteinuria was significantly higher than that in normal healthy subjects, and there was significantly positively correlated with the 24hUPro level of patients. ANGPTL3 might be involved in the formation of hyperlipidemia proteinuria.

## Abbreviations

24hUPro: 24 h urine protein quantitation
ALB: microalbumin
ANGPTL3: Angiopoietin-like protein 3
BUN: blood urea nitrogen
CKD: Chronic kidney disease
HDL: High density lipoprotein
HMGCoA-R: hydroxymethylglutaryl coenzyme A reductase
HTC: Hypercholesterolemia
HTG: Hypertriglyceridemia
K/DOQI: Kidney Disease Outcome Quality Initiative
LDL-C: Low density lipoprotein cholesterol
LPL: Lipoprotein lipase
NC group: Normal control group
SCr: Serum creatinine
TC: Cholesterol
TG: Triglyceride
UCL: University College London
VLDL: Very low density lipoprotein
